# Surgical Technique and Outcome of Custom Joint-Sparing Endoprosthesis as a Reconstructive Modality in Juxta-Articular Bone Sarcoma

**DOI:** 10.1155/2019/9417284

**Published:** 2019-12-26

**Authors:** Ahmad M. Shehadeh, Ula Isleem, Samer Abdelal, Hamza Salameh, Muthana Abdelhalim

**Affiliations:** ^1^Department of Orthopedic Oncology, King Hussein Cancer Center, Queen Rania Street, Amman, Jordan; ^2^Faculty of Medicine, University of Jordan, Queen Rania Street, Amman, Jordan

## Abstract

**Background:**

Joint-sparing limb salvage surgery (JSLSS) is an advancement in the techniques and concepts of limb salvage surgery, which makes it possible to save not only the limb affected by malignancy but also the adjacent joint and the epiphyseal plate. In the growing child, this procedure is technically demanding due to the availability of small length of bone for implant purchase. Reconstruction options can be biological reconstruction or endoprosthesis; however, the outcome of endoprosthetic reconstruction after joint-sparing resection is not well described in the literature.

**Purposes:**

(1) To determine the prosthesis survival rates when using customized Joint-Sparing Endoprosthesis (JSE) after juxta-articular resection of bone tumors, (2) to investigate the rates of local recurrence, (3) to evaluate the need for revision surgery, and (4) to compare the outcome of customized JSE with that of joint-sacrificing techniques.

**Methods:**

In our study, joint sparing is defined as any procedure where a custom-made JSE is used in lieu of sacrificing the adjacent joint whenever the length of the remaining bone segment is not enough to accommodate the stem of a modular implant. Twenty-eight patients received JSE, and 31 joints were spared. Their age ranged from 4 to 55 years with a median age of 13 years. Twenty-one patients received surgery for primary reconstruction and 7 patients for revision of failed bone allograft or modular implant. Twenty-four joints are spared in the lower limbs and 7 in the upper limbs. Osteosarcoma was the most common pathological diagnosis (*n* = 13). Flat surface HA-coated custom JSE was used to spare 15 joints, and short-stemmed custom JSE was used to spare 16 joints. The length of the remaining bone epiphysis for JSE anchorage from the knee and ankle joints was 25–75 mm, median = 45 mm, and the length of the cortical bone remaining for the proximal femur and distal humerus was 5–70 mm, median = 10 mm.

**Results:**

Operative time was 2.5 to 4 hours (avg. 3 hr.) The bone resection surface fitted the prosthesis surface with <2 mm difference. Histological examination of all resected specimens shows clear bone resection margins; 2 patients had positive soft tissue margins. At mean follow-up period of 3 years (6 months–10 years), 6 patients developed local and systemic recurrences, three of them had a pathological fracture at the time of diagnosis (*P*=0.139), and 4 showed a poor response to chemotherapy (*P*=0.139), and 4 showed a poor response to chemotherapy (

**Conclusions:**

Whenever this kind of implant is affordable and can be utilized, particularly in younger age groups, JSE may be a good reconstruction option to avoid the use of expandable implants and to avoid the potentially higher revision and complication rates associated with biological reconstruction, as well as the complications of conventional joint-sacrificing implant, mainly dislocations and polyethylene wear and tear.

## 1. Introduction

Joint-sparing limb salvage surgery (JSLSS) is defined as the retention of the native joint in adults and the epiphyseal plate in children when resecting a juxta-articular bone sarcoma. Most patients of bone sarcoma are adolescents and young adults [1]. Furthermore, there are longer survival rates associated with current multidisciplinary treatment modalities. These factors increase the importance of limb and joint salvage in these patients.

Sparing the native joints and epiphyseal plate has several advantages. Preservation of the native joint and all attached ligaments can lead to better proprioception [2], fewer complications related to polyethylene liners of the artificial joints, possible preservation of growth potential, and a decreased need for expandable implants. Preservation of important tendinous attachments can also facilitate rehabilitation and subsequent function.

The challenge in these patients has been to use construct that can reliably grip and hold the small joint- or physis-containing fragment [3]. Reconstruction options after joint-sparing resection of juxta-articular bone sarcoma can be biological (bone allograft and autograft) versus customized joint-sparing implants. The outcome of reconstruction using custom-made Joint-Sparing Endoprosthesis (JSE) is not well described in the literature. A thorough review of the literature shows that only a few papers discussed the outcome of using custom-made implants, with a handful number of patients in each [3–6]. In this paper, we investigate the outcome of using custom JSE in 28 patients and 31 joints, the largest series investigating the outcome of custom JSE in the literature, so far.

The purposes of this study, therefore, were (1) to determine the 5-year survival when using customized Joint-Sparing Endoprosthesis (JSE) after juxta-articular resection of bone tumors, (2) to investigate the rates of local recurrence, (3) to evaluate the need for revision surgery, and (4) to compare the outcome of customized JSE with that of joint-sacrificing techniques.

## 2. Methods

This retrospective study was conducted at the King Hussein Cancer Center, the only comprehensive cancer center in Jordan.

In our study, joint sparing is defined as any procedure where a custom-made JSE is used in lieu of sacrificing the adjacent joint, in cases where the remaining bone after tumor resection cannot accommodate the stem of a modular implant.

As a reconstructive modality for the resected diaphyseal-metaphyseal segment of bone, we used custom-made JSE, which can have one of the two following designs.

**Figure 1:**
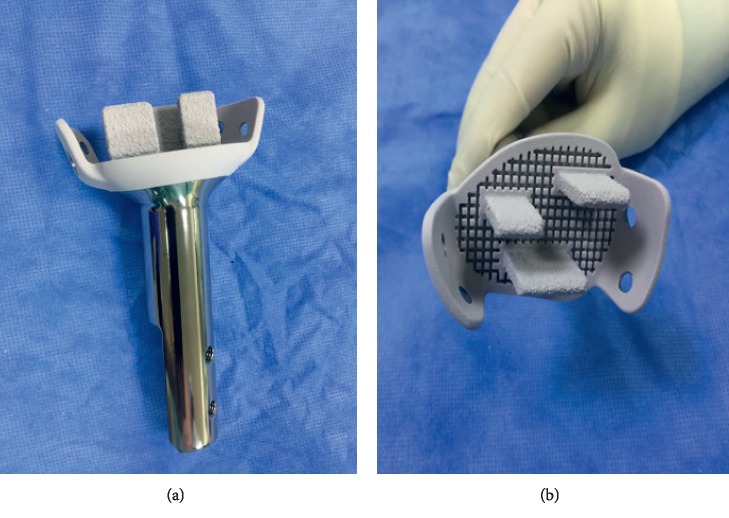
Flat surface custom JSE with 3 HA-coated fins and 2 side plates.

The first deign is flat surface hydroxyapatite- (HA-) coated titanium implants, which corresponds neatly to the dimensions and shape of the metaphyseal-epiphyseal residual bone surface. This design is used whenever we have less than 3 cm of cortical bone remaining, and the fixation will be based mainly on the metaphyseal-epiphyseal bone segment. These implants are provided with 2 to 3 HA-coated, fenestrated fins to maximize bone integration into the metal surface and act as anchorage tool of the implant to the remaining bone segment. These designs are always cementless. They are also equipped with 2-3 HA-coated side plates that are important to prevent angulation while bone ingrowth is still in progress. Construct stability will depend on the successful osseointegration between the implant and the remaining bone (Figure 1).

**Figure 2:**
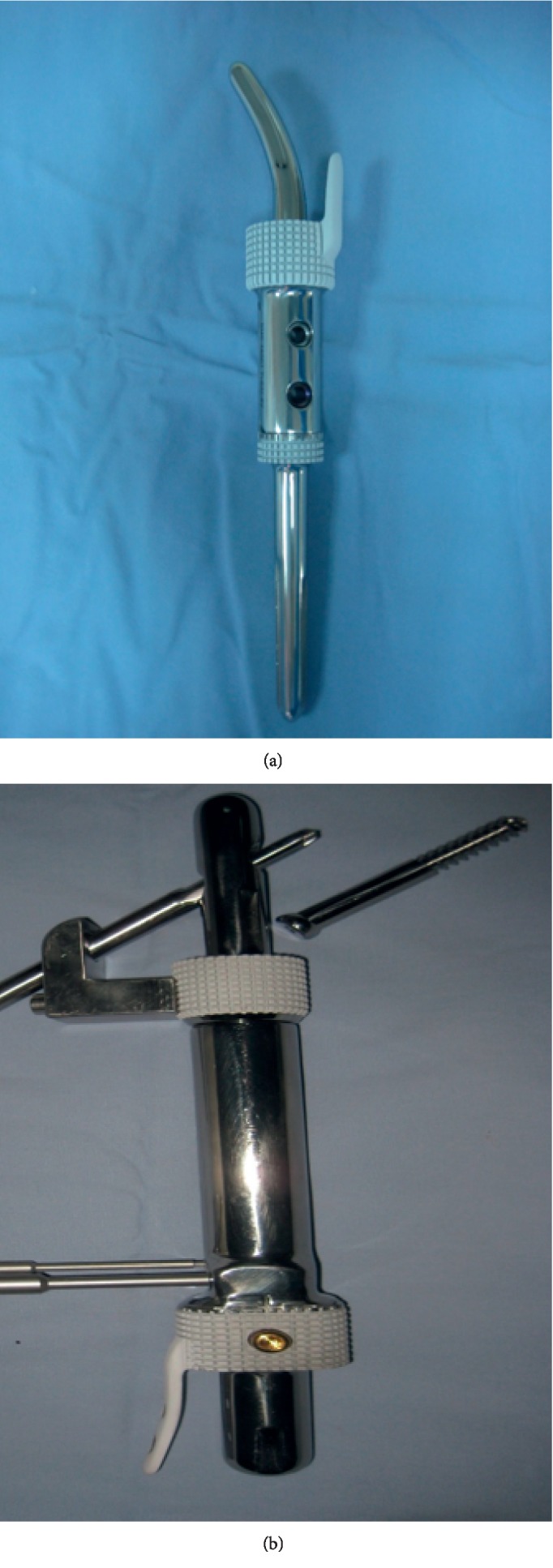
(a) Custom JSE with curved stem and HA-coated side plate suitable for proximal femur. (b) Custom JSE for proximal femur and distal femur with cross screw, and one side plate for the distal femur side.

The second design is cemented short-stemmed implants, where the stem can be either a straight short stem with cross screw(s) or a curved banana shaped stem in proximal femur cases. This design is utilized in cases where the residual cortical bone (diaphyseal bone) is at least 3 cm in length. Both of these are provided with HA-coated side plate(s) that will prevent rotation and stem toggling, and all these stems are fixed with bone cement (Figure 2).

**Figure 3:**
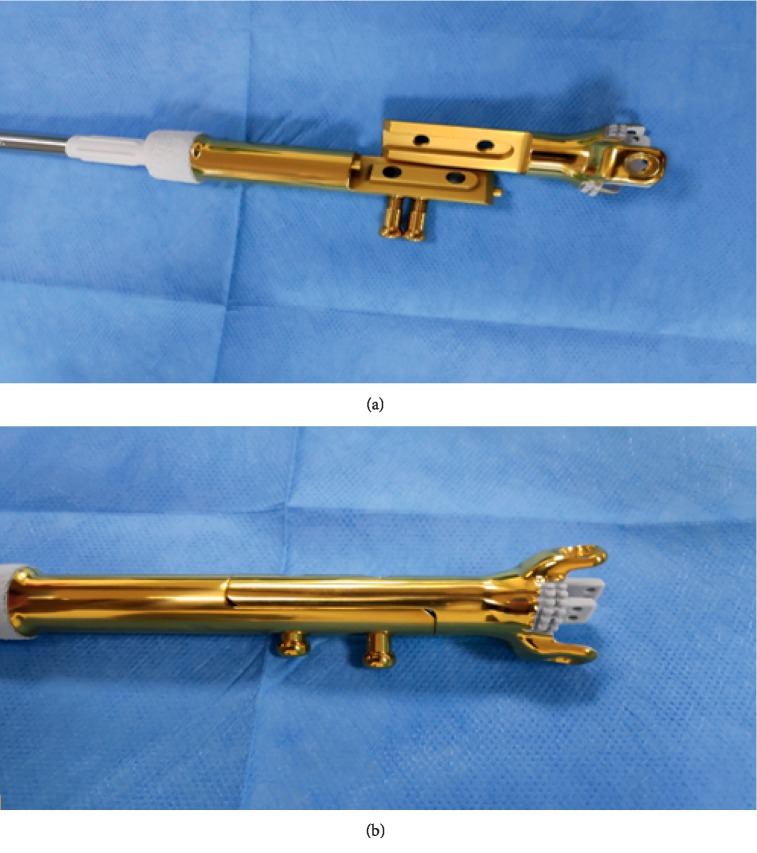
The 2 parts of the JSE and 2 connecting screws. (a) Before assembly. (b) After assembly.

The other side of both implants is fixed to the diaphyseal bone by a cemented or cementless stem; all implants come in 2 pieces: one piece fixed to the metaphyseal-epiphyseal segment of bone, and the other piece fixed to the tubular diaphysis side. Next, both pieces are connected to each other by male-female side mechanism and locked with 2 screws (Figure 3).

### 2.1. Surgical Protocol

**Figure 4:**
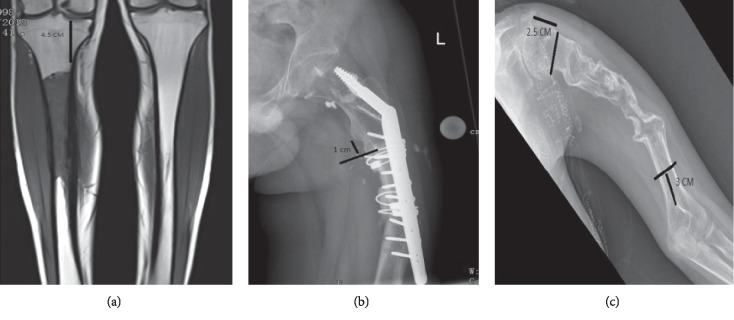
(a) MRI of right tibia of patient with a tibial osteosarcoma, with 4.5 cm remaining length for anchorage of JSE after resection of the tumor with negative margin. (b) Left proximal femur X-ray of a failed bone allograft and DHS cut-off, with 1 cm of cortical bone remaining below the lesser trochanter. A JSE can be used here to revise this failed bone allograft. (c) Left humerus X-ray of postchemotherapy humerus osteosarcoma, showing a 3 cm of cortical bone remaining above the olecranon fossa which makes the sparing of the elbow joint possible. Furthermore, 2.5 cm of bone is remaining proximally making the sparing of the glenohumeral joint possible.

Magnetic resonance images and computerized tomography scans for the affected limb are reviewed thoroughly and carefully by the operating surgeon and radiologists. All distal femurs, proximal tibias, distal tibias, and proximal humeri with a residual bone of more than 25 mm from the articular surface, after tumor resection with at least a 1.5 cm safety margin, were considered eligible to receive joint-sparing resection and JSE reconstruction (Figure 4(a)). All proximal femur segments with 5 mm or more cortical subtrochanteric bone remaining after tumor resection with at least a 1.5 cm safety margin were considered eligible for proximal femur JSE, and all distal humerus segments with 3 cm or more remaining cortical bone above the olecranon fossa were considered eligible for distal humerus JSE (Figures 4(b) and 4(c)).

**Figure 5:**
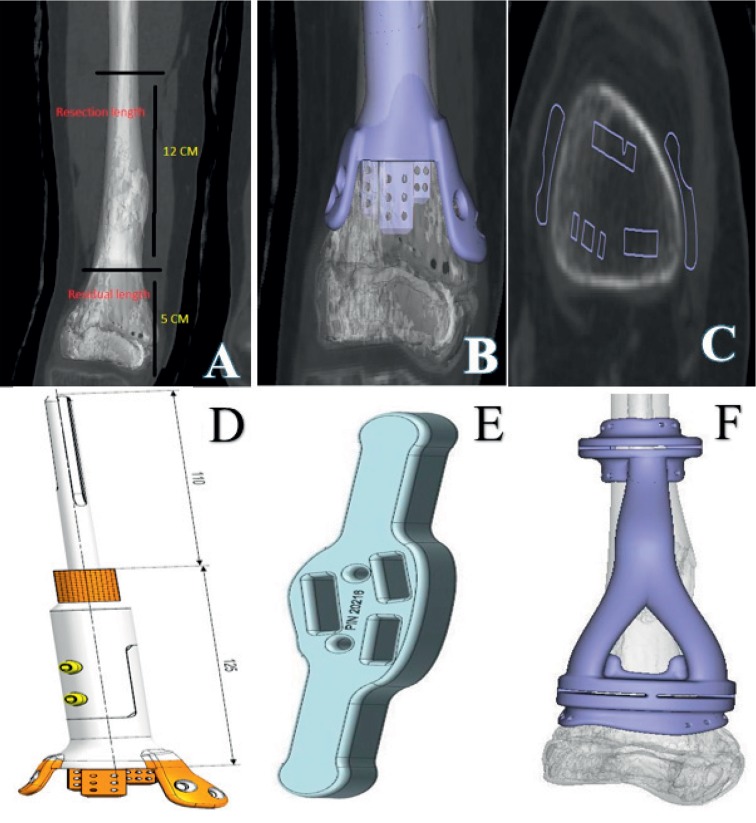
(a) A measuring CT scan of femur showing the resection length and residual metaphyseal-epiphyseal bone segment length. (b, c) Fitting of the implant design into the residual bone segment. (d) The final JSE design. (e) The 3D printed fin template which will match in size and dimensions to the metaphyseal surface of residual bone. (f) The 3D printed cutting guide which will be used to make distal and proximal bone osteotomies.

The MRI and CT scans are sent to the manufacturer (Stanmore Implants Worldwide Ltd, Middlesex, UK) along with our measurements for resection and residual bone length, as well as our design concepts (Figure 5). Afterward, we receive the design proposal for review. If no modifications are needed, then we approve the proposal and wait 6 weeks to have the implant delivered to our hospital.

### 2.2. Surgical Technique

**Figure 6:**
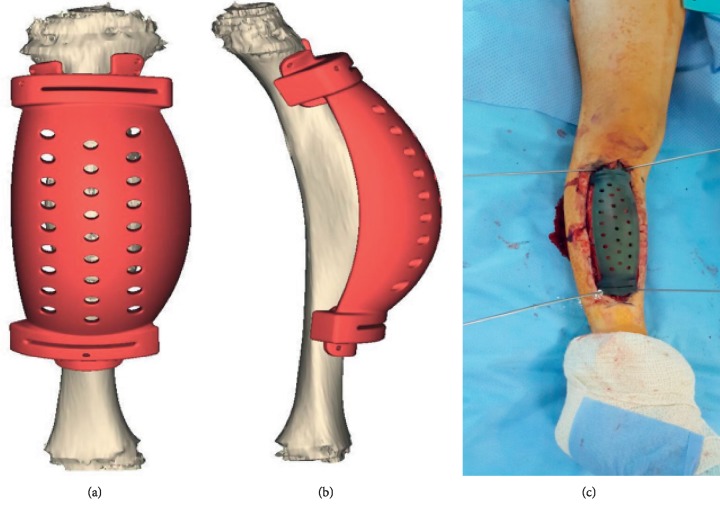
(a, b) The 3D printed cutting guide for tibia. (c) The fixation of the 3D printed cutting guide to the patient's tibia using k-wires.

After general anesthesia, positioning of the patient and prophylactic antibiotics are given. The skin incision is made, creating two flaps. After the release of soft tissue attachments, as well as identification and protection of related major neurovascular structures, a 3D printed cutting guide is held to the surface of the bone harboring the tumor (Figure 6).

**Figure 7:**
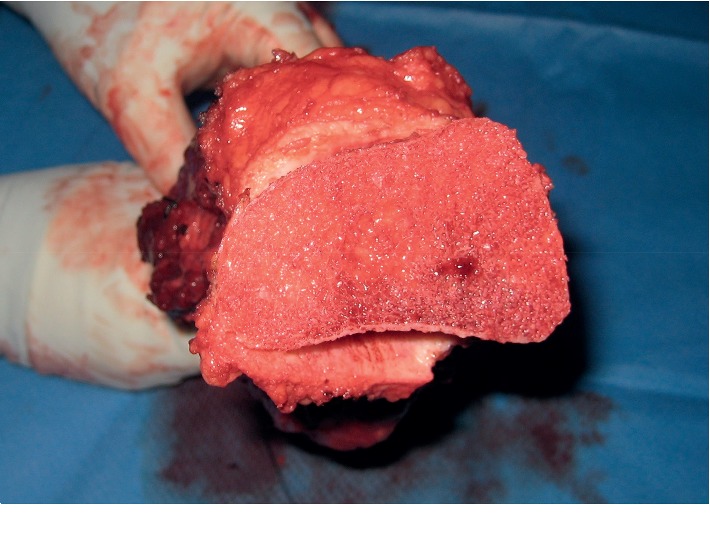
The resection specimen showing the metaphyseal cut surface.

Proximal and distal osteotomies through the cutting guide slots are performed. After harvesting bone marrow tissue samples from the proximal and distal bone margin, they are sent for frozen section. Next, we completely resect the specimen (Figure 7).

**Figure 8:**
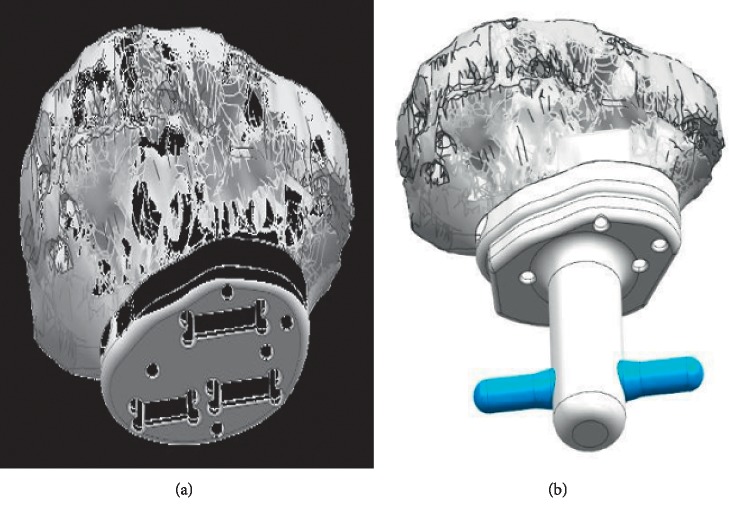
(a) Diagram showing the application of fin template to the residual bone surface. (b) The use of the impaction tool to prepare the fin tracks.

**Figure 9:**
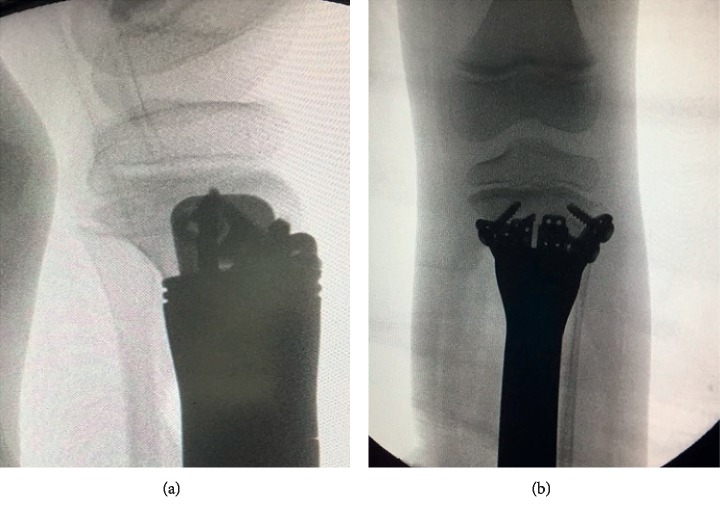
Intraoperative image intensifier for the position of the implant in the metaphyseal bone segment. (a) Lateral view. (b) AP view.

Preparation of the metaphyseal-epiphyseal bone segment is done using a fin template guide and an impaction tool under image intensifier control (Figure 8). After implantation of the joint-saving endoprosthesis, we check the position again under image control and then fix the implant to the epiphyseal bone segment with screws through the side plate (Figure 9).

**Figure 10:**
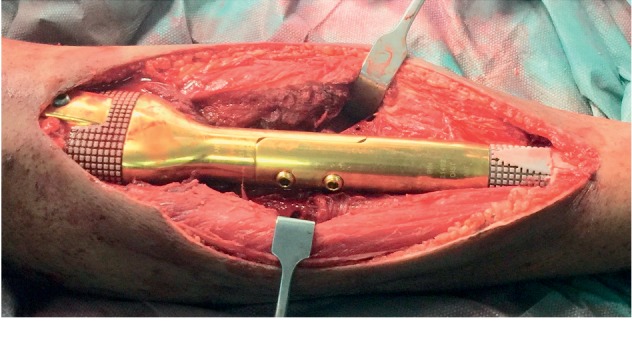
Completion of assembly of the 2 sides of the JSE using 2 connecting screws.

The second piece of the implant is fixed to the other side of the host bone using a cemented or cementless stem. Finally, both parts of the implant are assembled using locking screws (Figure 10).

Soft tissue coverage of the implant is performed, drains are inserted, and then the incision is closed in layers. Splinting using back slabs is recommended to provide extra stability to the construct and prevent the patient from premature weight bearing.

### 2.3. Rehabilitation and Aftercare

In all patients with flat surface JSE, where the fixation mechanism is based on successful osseointegration between the implant and the bone, the operative limb is kept in an external splint and protected from weight bearing for 8–12 weeks. In patients with a short-cemented stem, they started full weight bearing, as tolerated, at first day postoperatively.

## 3. Results

A total of 28 patients received custom JSE, 21 of them for primary reconstruction and 7 patients for the revision of a failed bone allograft or modular implant. Thirty-one joints were spared: distal femur (*n* = 9), proximal tibia (*n* = 8), proximal femur (*n* = 6), distal humerus (*n* = 4), proximal humerus (*n* = 2), and distal tibia (*n* = 2).

Of the 7 patients who received JSE for revision, 2 of them had limb-length discrepancy, which prompted the use of an expandable JSE. The expansion was successfully performed at 5 cm and 9 cm for the two patients. The ages of the patients ranged from 4 to 55 years, with a median age of 13 years. Osteosarcoma was the commonest histological diagnosis Table 1.

### 3.1. Survival Rates and Complications of the Custom Joint-Sparing Endoprosthesis

Operative time was found to be 2.5–4 hours (with an average of 3 hours). The bone resection surface was fitted to the prosthesis surface with a difference of <2 mm in all dimensions, indicating accuracy of the osteotomies' level. All bone resection margins were free of tumor. Two patients had tumor-positive soft tissue margins.

**Figure 11:**
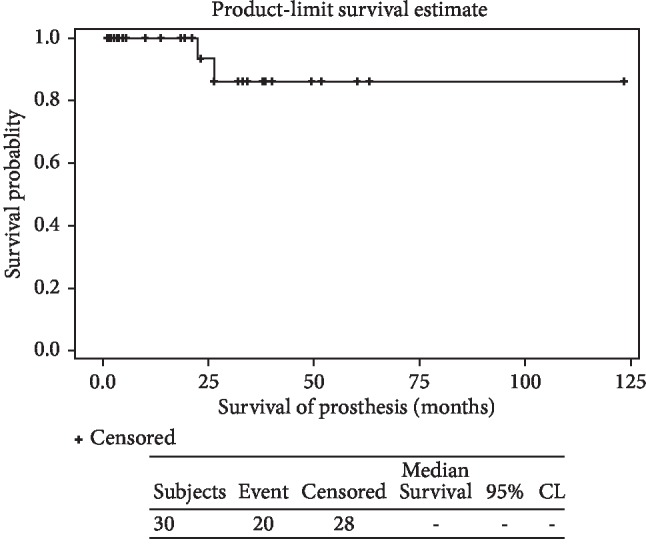
The Kaplan–Meier survival curve analysis of all 28 JSE used: the 3-year survival rate: 86.15%; 5-year survival rate: 86.15%.

Implant survival at 3 and 5 years was 88.44 and 86.15%, respectively. (Figure 11).

**Figure 12:**
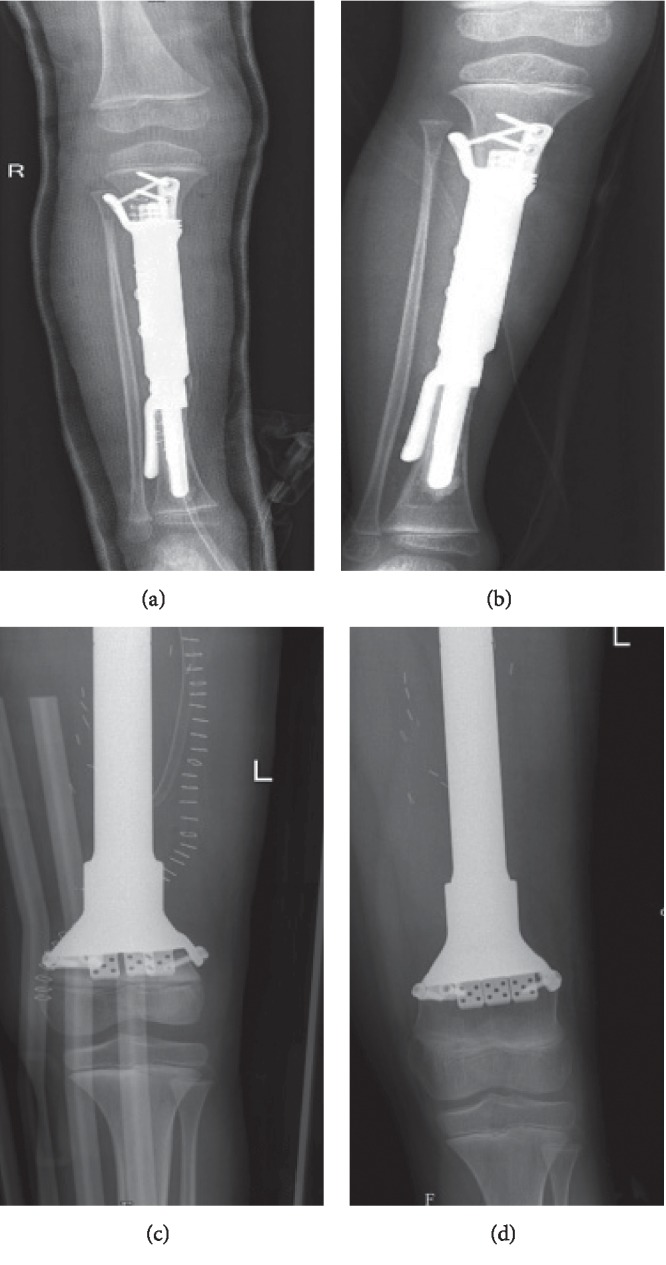
(a) Proximal tibia JSE immediately postoperatively and (b) one year after surgery showing the growth of the proximal tibia segment. (c) Distal femur JSE immediately postoperatively and (d) one year after surgery, showing the growth of the distal femur segment.

Twenty-seven of the joints did well with no further operative intervention. Growth of the remaining epiphyseal segment was observed in serial radiological follow-up in all 6 skeletally immature patients who received JSE around the knee joint, with no limb-length discrepancy (Figure 12).

**Figure 13:**
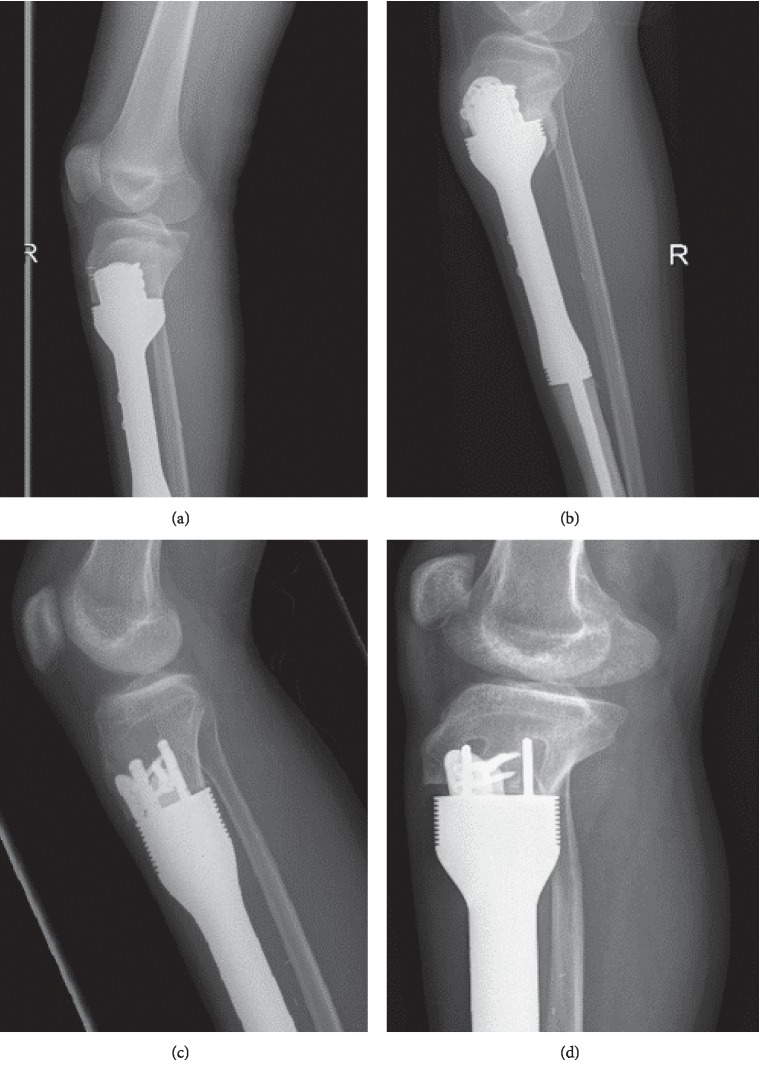
Proximal tibia JSE (a) immediately after surgery and (b) 8 months later, with tilting of the proximal metaphyseal tibia segment, and extra cortical bone formation indicating loosening of the implant and failure of osseointegration. (c) Proximal tibia JSE in another patient immediate after surgery and (d) 6 months later, with tilting of the proximal metaphyseal tibia segment, and radiolucent pockets around the fins, indicating failure of osseointegration mechanism.

**Figure 14:**
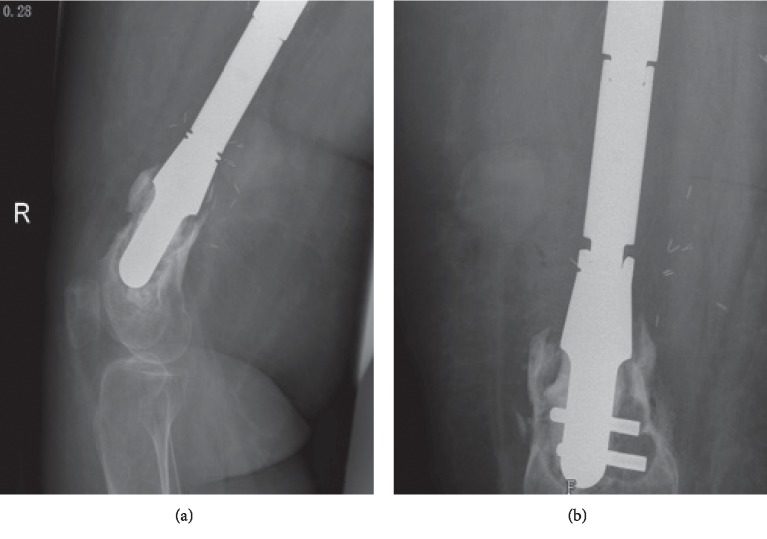
(a) Lateral and (b) AP films of distal femur cemented JSE, 2 years after surgery, showing radiolucent lines and bone destruction of the remaining bone segment.

Two patients with flat surface JSE developed failure of osseointegration and loosening of the implant, both with proximal tibia JSE, 4 and 6 months postoperatively, respectively. Clinically, the patients presented with pain on weight bearing. Radiologically, their X-rays show angulation of the implant with a radiolucent line around the fins. Both of them received revision surgery with a new flat surface JSE and did well at the last follow-up (Figure 13). One patient with a cemented short stem JSE of distal femur, developed loosening but died of systemic disease before revision (Figure 14). The median-modified MSTS score measured 6–12 months after surgery was 90% (83–96%). None of the patients developed joint contractures.

### 3.2. Local Recurrence Rates of the Tumor

Out of the 24 patients (21 were reconstructed with JSE, and 3 with bone allograft and revised later with JSE) who received joint-sparing surgery for primary tumor reconstruction, 6 developed local recurrences (25%). All recurrences occurred in the soft tissue, with none occurring in the residual bone segment. Three of those with local recurrence had pathological fractures (*P*=0.139) and 4 showed a poor response to chemotherapy (*P*=0.014). One of them had positive soft tissue margin (*P*=0.446) and 4 of them had systemic recurrence Table 2.

Two of them were treated with amputation, two received resection of the soft tissue mass, and two died of systemic recurrence and did not receive surgery for the local recurrence.

**Figure 15:**
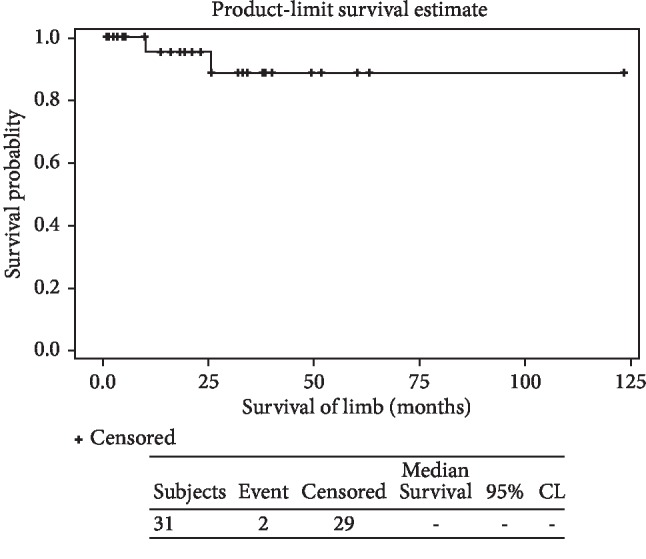
The Kaplan–Meier survival analysis of all 28 limbs: the 3-year survival rate was 88.44% and the 5-year survival rate was 86.15%.

Limb survival was 88.44% at 3 years and 86.15% at 5 years (Figure 15).

### 3.3. Periprosthetic Fracture

One patient with proximal tibia JSE sustained a periprosthetic fracture at the distal cemented stem and received fixation with plate and screws (Figure 14).

## 4. Discussion

Joint sparing has many advantages as mentioned previously; however, it also comes with many challenges. The main challenge is finding the appropriate reconstruction modality. A thorough review of the literature shows that most of the case series employed biological reconstruction. The outcome of biological reconstruction is associated with a higher incidence of failure, due to fracture, nonunion, and infection, in addition to the long operative time needed, especially when utilizing vascularized fibulas. There was also a frequent need for revision surgery as well as, prolonged protected weight bearing after surgery and longer rehabilitation. The incidence of all major complications encountered in biological reconstruction ranges from 32 to 47% [2, 7–13], with similar revision rates. Furthermore, in many techniques used in bone allograft reconstruction, the fixation plate will cause premature epiphysiodesis and early closure of the epiphyseal plate, with no attempt at preserving the growth potential of the growing bone and subsequent need for bone elongation procedures Table 3 [7].

The use of custom-made JSE is previously described in the literature [3, 4]. The complications associated with this approach were deep infection and periprosthetic fractures, but no limb loss for implant-related complications; however, the number of patients included in previously reported papers regarding JSE is relatively small.

In this study, we report the outcome of 28 patients receiving custom JSE, the biggest series on this topic, to date.

Three of our patients developed implant-related complications and 6 developed local recurrences, which was found to be related significantly to poor response to chemotherapy in the subgroup of patients who received this surgery, rather than the surgery itself (*P* < 0.05).

When comparing the complications and the revision rate in this series to others using a bone allograft, a lower complication rate was found and operative time was shown to be shorter (Table 4). When compared to joint-sacrificing approaches, there was no increased incidence of local recurrence or implant-related complications. Furthermore, the functional outcome was found to be favorable, with our study showing an MSTS score of 90%.

After reviewing the survival rates and complications of conventional joint-sacrificing endoprosthesis, Shehadeh et al. [14] studied the outcome of endoprosthesis in 232 patients. The total complication rate was 41%, the revision rate of the implants was 29%, and the infection rate was 13%. The 5-year survival of modular and custom-made implants was 85% and 79%, respectively. Comparing our results to those results shows a favorable outcome, with a 5-year survival rate of 86% in our study.

One of the limitations of this study is the fact that this is a retrospective study over a relatively short duration. Although this study has the largest sample size, the patient population is still too small to draw any firm conclusions or to compare outcomes between different anatomical locations. In addition, there is no comparison with control groups with biological reconstruction and conventional joint-sacrificing approach.

Nonetheless, our study outcomes show that the use of JSE in joint-sparing limb salvage surgery is a safe reconstruction modality with no increased risk of complications and does not jeopardize the oncological principles for bone tumors surgery.

## 5. Conclusion

Whenever this kind of implant is affordable and can be utilized, particularly in younger age groups, JSE may be a good reconstruction option to avoid the use of expandable implants and to avoid the potentially higher revision and complication rates associated with biological reconstruction, as well as the complications of conventional joint-sacrificing implant, mainly dislocations and polyethylene wear and tear.

## Figures and Tables

**Table 1:** The histological diagnosis of all patients in this study.

Histological diagnosis	Number of patients
Osteosarcoma	13
Ewing sarcoma	10
Adamantinoma	3
Gorham's disease	1
Myoepithelial carcinoma	1

**Table 2:** The association between tumor recurrence and response to chemotherapy, pathological fracture, and surgical margins.

Factors	Presence/absence	Total	Local recurrence		*P* value
			Absent	Present	
Pathologic fracture	No	18 (75.0%)	15 (83.3%)	3 (50.0%)	0.139
	Yes	6 (25.0%)	3 (16.7%)	3 (50.0%)	
Response to neoadjuvant chemotherapy (*n* = 21)	Good response	14 (70.0%)	13 (86.7%)	1 (20.0%)	0.014
	Poor response	6 (30.0%)	2 (13.3%)	4 (80.0%)	
Soft tissue margins	Negative	22 (91.7%)	17 (94.4%)	5 (83.3%)	0.446
	Positive	2 (8.3%)	1 (5.6%)	1 (16.7%)	

**Table 3:** The literature review of joint-sparing limb salvage using biological reconstruction, showing complications and functional outcome.

Series	Year	No. of patients	Implant/allograft and site of the tumor	Survival of the allograft at last follow-up	Complications/revision	Infection	Recurrence	MSTS score
(1) Manfrini et al. [12]	1999	10	Vascularized fibula graft and massive allograft		Valgus deformity limb-length discrepancy 2–3.5 cm	None reported	None reported, but 4 were lost for follow-up	Satisfactory
(2) Aponte-Tinao et al. [7]	2015	35	Epiphyseal preservation and allograft tibia and femur	Overall survival rate of the patients was 86% at 5 years	14 (40%) patients Fracture and nonunion. Removal in 10 patients	2 (6%)	3	The mean functional score was 26 points at final follow-up
(3) Agarwal et al. [3]	2010	19	Bone allograft, autograft, or vascularized fibula	16/25 at a median follow-up of 34 months. There were four deaths	4 (22%) fracture and nonunion	2 (10%)	4	The musculoskeletal tumor society score ranged from 27 to 30
(4) Abdelaal et al. [9]	2015	18	Epiphyseal sparing and reconstruction	Five- and ten-year rates of survival were 94.4%	Fracture of the graft and nonunion in 2 patients (11%)	2 (11%)	1	MSTS score was excellent in 17 patients (94.4%) and poor in one (5.5%)
(5) Li et al. [11]	2017	41	Vascularized fibula and bone allograft	All at follow-up of 3–11 years Mean 4.4 years	Revision in 10 patients (24%) osteonecrosis in remaining epiphysis in 13 patients. (31%)		3	MSTS score 22–30 Median = 28
(6) Muratori et al. [13]	2018	64	Resections around ankle, knee, and hip	Mean follow-up was 117 months (12–305)	Fracture 26%. Nonunion 14%	3 patients (4.7%)	Not reported	27 (18–30)

**Table 4:** The literature review of joint-sparing limb salvage using JSE for reconstruction, showing complications and functional outcome.

Series	Year	No. of patients	Implant/allograft and site of the tumor	5 year survival of the implant or the allograft or last follow-up	Complications/revision	Infection	Recurrence	MSTS score
(1) Gupta et al. [4]	2006	8	Knee-sparing distal femoral endoprosthesis	Mean follow-up: 24 months	0	1 patient developed septicemia two weeks after surgery	0	The mean was 80% (57% to 96.7%)
(2) Agarwal et al. [3]	2010	6	Custom implant prosthesis	Mean follow-up: 12–27 months 1 patient died	0	2 deep infection	1	The score ranged from 27 to 30
(3) Wong et al. [6]	2013	8	6 femur, 1 tibia, and 1 proximal humerus	Mean follow-up: 41 months	0	0	0	The mean score was 29.1 (range, 28–30)
(4) Spiegelberg et al. [5]	2009	8	Stanmore proximal tibia replacement epiphyseal sparing	The mean follow-up: 35 months	1 periprosthetic fracture	0	1 converted to BKA	The mean score was 24 of 30 79% (57% to 90%)
This study	2019	28	Custom Joint-Sparing Endoprosthesis (JSE), from Stanmore	The mean follow-up: 3 years	3 loosening 2 of them revised with new JSE	0	6, 2 of them received AKA	The mean score was 90% (83–96%)

## Data Availability

The data used to support the findings of this study are included within the article.

## References

[1] Burningham Z., Hashibe M., Spector L., Schiffman J. D. (2012). The epidemiology of sarcoma. *Clinical Sarcoma Research*.

[2] Kumta S. M., Chow T. C., Griffith J., Li C. K., Kew J., Leung P. C. (1999). Classifying the location of osteosarcoma with reference to the epiphyseal plate helps determine the optimal skeletal resection in limb salvage procedure. *Archives of Orthopaedic and Trauma Surgery*.

[3] Agarwal M., Puri A., Gulia A., Reddy K. (2010). Joint-sparing or physeal-sparing diaphyseal resections: the challenge of holding small fragments. *Clinical Orthopaedics and Related Research*.

[4] Gupta A., Pollock R., Cannon S. R., Briggs T. W. R., Skinner J., Blunn G. (2006). A knee-sparing distal femoral endoprosthesis using hydroxyapatite-coated extracortical plates. *The Journal of Bone and Joint Surgery. British Volume*.

[5] Spiegelberg B. G. I., Sewell M. D., Aston W. J. S. (2009). The early results of joint-sparing proximal tibial replacement for primary bone tumours, using extracortical plate fixation. *The Journal of Bone and Joint Surgery. British Volume*.

[6] Wong K. C., Kumta S. M. (2013). Joint-preserving tumor resection and reconstruction using image-guided computer navigation. *Clinical Orthopaedics and Related Research*.

[7] Aponte-Tinao L., Ayerza M. A., Muscolo D. L., Farfalli G. L. (2015). Survival, recurrence, and function after epiphyseal preservation and allograft reconstruction in osteosarcoma of the knee. *Clinical Orthopaedics and Related Research®*.

[8] Avedian R. S., Haydon R. C., Peabody T. D. (2010). Multiplanar osteotomy with limited wide margins: a tissue preserving surgical technique for high-grade bone sarcomas. *Clinical Orthopaedics and Related Research®*.

[9] Hamed Kassem Abdelaal A., Yamamoto N., Hayashi K., Takeuchi A., Miwa S., Tsuchiya H. (2015). Epiphyseal sparing and reconstruction by frozen bone autograft after malignant bone tumor resection in children. *Sarcoma*.

[10] Li J., Shi L., Chen G.-J. (2014). Image navigation assisted joint-saving surgery for treatment of bone sarcoma around knee in skeletally immature patients. *Surgical Oncology*.

[11] Li J., Wang Z., Ji C., Chen G., Liu D., Zhu H. (2017). What are the oncologic and functional outcomes after joint salvage resections for juxtaarticular osteosarcoma about the knee?. *Clinical Orthopaedics and Related Research®*.

[12] Manfrini M., Gasbarrini A., Malaguti C. (1999). Intraepiphyseal resection of the proximal tibia and its impact on lower limb growth. *Clinical Orthopaedics and Related Research*.

[13] Muratori F., Totti F., D’Arienzo A. (2018). Biological intercalary reconstruction with bone grafts after joint-sparing resection of the lower limb: is this an effective and durable solution for joint preservation?. *Surgical technology international*.

[14] Shehadeh A., Noveau J., Malawer M., Henshaw R. (2010). Late complications and survival of endoprosthetic reconstruction after resection of bone tumors. *Clinical Orthopaedics and Related Research®*.

